# Evaluation of Four Artificial Intelligence–Assisted Self-Diagnosis Apps on Three Diagnoses: Two-Year Follow-Up Study

**DOI:** 10.2196/18097

**Published:** 2020-12-04

**Authors:** Aleksandar Ćirković

**Keywords:** artificial intelligence, machine learning, mobile apps, medical diagnosis, mHealth

## Abstract

**Background:**

Consumer-oriented mobile self-diagnosis apps have been developed using undisclosed algorithms, presumably based on machine learning and other artificial intelligence (AI) technologies. The US Food and Drug Administration now discerns apps with learning AI algorithms from those with stable ones and treats the former as medical devices. To the author’s knowledge, no self-diagnosis app testing has been performed in the field of ophthalmology so far.

**Objective:**

The objective of this study was to test apps that were previously mentioned in the scientific literature on a set of diagnoses in a deliberate time interval, comparing the results and looking for differences that hint at “nonlocked” learning algorithms.

**Methods:**

Four apps from the literature were chosen (Ada, Babylon, Buoy, and Your.MD). A set of three ophthalmology diagnoses (glaucoma, retinal tear, dry eye syndrome) representing three levels of urgency was used to simultaneously test the apps’ diagnostic efficiency and treatment recommendations in this specialty. Two years was the chosen time interval between the tests (2018 and 2020). Scores were awarded by one evaluating physician using a defined scheme.

**Results:**

Two apps (Ada and Your.MD) received significantly higher scores than the other two. All apps either worsened in their results between 2018 and 2020 or remained unchanged at a low level. The variation in the results over time indicates “nonlocked” learning algorithms using AI technologies. None of the apps provided correct diagnoses and treatment recommendations for all three diagnoses in 2020. Two apps (Babylon and Your.MD) asked significantly fewer questions than the other two (*P*<.001).

**Conclusions:**

“Nonlocked” algorithms are used by self-diagnosis apps. The diagnostic efficiency of the tested apps seems to worsen over time, with some apps being more capable than others. Systematic studies on a wider scale are necessary for health care providers and patients to correctly assess the safety and efficacy of such apps and for correct classification by health care regulating authorities.

## Introduction

Algorithms and machine learning (ML) have conquered, transformed, and essentially revolutionized people’s everyday lives in many aspects (eg, with personalized Google searches, self-driving cars, or convenient smartphone apps) [1,2]. In recent years, self-diagnosis apps have emerged that allow patients to look for a diagnosis based on entered symptoms [3,4]. ML is already being standardly used in various applications like estimating diagnoses from radiology images [5], but the adoption and acceptance of new technologies in health care in general is curbed by trust issues, strict regulations, and lack of thorough investigation [3,6]. Little testing of the aforementioned apps has been previously performed; Semigran et al tested self-diagnosis apps in general in 2015 but did not mention ML as an underlying technology [7]. A recent scoping review by Aboueid et al in 2019 named several apps of this type [3], but only two have been tested in their diagnostic functionality so far [8-10]. The US Food and Drug Administration (FDA) had previously excluded “symptom checker” apps from the enforcement of the strict rules that are usually applied to medical devices [11] but has lately released a white paper with a proposal for possible changes in the regulation of self-diagnosis apps, introducing a new discrimination between “locked” and artificial intelligence (AI)–based/ML-based learning algorithms, with the latter falling under a stricter set of rules [12]. However, the provided definitions of the two categories are still vague and rely on the manufacturer admitting the use of AI-based technologies rather than on hard criteria. Repeated examinations of such apps would offer insight into whether the results they offer are stable over time or change, which would also indicate actual use of “learning” AI technologies by the manufacturers, rendering the apps more than plain symptom checkers. Thus, the first aim of this study was to test apps that have previously been mentioned in the scientific literature on a set of diagnoses and to subsequently follow up on the results after a deliberate time interval to determine whether the algorithms change over time. A significant change in the results might indicate that learning algorithms are used by the manufacturers.

None of the aforementioned studies had tested self-diagnosis apps in the field of ophthalmology, the author’s primary specialty with over 12 years of clinical experience. Thus, the second aim of the study was to test the efficacy of self-diagnosis apps by presenting them with three common diagnoses representing three levels of urgency in this field to see if their results and treatment recommendations match those of someone familiar with the topic.

## Methods

### Overview

The mobile apps were tested on Android 9 and Android 10, and the web app was tested on Google Chrome for OSX. All tests took place in Germany, and in all cases the English user interfaces were used. For all apps, up-to-date versions in the Google Play Store or on the internet were used; they correspond to the dates noted for each of the diagnostic walk-throughs (see Multimedia Appendix 1). The programs that were included in the testing were those that have been mentioned in the scientific literature: Ada [3,8,13], Babylon Health or Babylon Check [3,9,13], Buoy Health [14], and Your.MD [13]. Excluded from testing were Baidu Doctor [15], which is available only in Chinese, and K Health [3], which could not be downloaded onto the devices due to regional unavailability on iOS and unresolvable compatibility issues on Android, which may also stem from regional restriction without explicit notification. While there are reports about Buoy that claim that an Android-based app is in development, at the time of testing it could only be used as a web app [16]. The basic functional principles as understood by the author or described by the software developers themselves and recent literature, including gray literature where scientific literature was unavailable, were summarized. There is no consensus on broad testing of AI-based apps yet, but in the past, symptom checker apps have been tested using a randomized set of virtual diagnoses combined with gathered patient information in sets called vignettes [7]. The same setup has been used recently in a 2018 study by Razzaki et al to test Babylon Health [10] and in a 2019 study by Jungmann et al to test Ada Health in the field of mental disorders [8]. Only one physician creating one virtual patient per diagnosis was involved in this work; thus, an abbreviated and simplified version of this procedure was performed as follows. Three defined diagnoses from the ophthalmology branch of medicine were entered via the apps’ given user interfaces: representing an absolute emergency (immediate treatment recommended), a glaucoma attack in one eye with the typical combination of a painful red eye for about two hours, blurred vision, a headache, and other symptoms depending on how each app asked its questions (see Multimedia Appendix 1 for the walk-through of all diagnoses as experienced); as a relative emergency (same-day treatment recommended), a retinal tear; and as a diagnosis that does not need immediate treatment and may also be self-treated first, dry eyes. For all three diagnoses, there are no strict general clinical therapy guidelines available, but some regional societies offer general recommendations [17-19]. The symptoms of all three diagnoses are common knowledge in ophthalmology and are thoroughly examined in the American Academy of Ophthalmology’s Basic and Clinical Science Course, congruent with the author’s applied knowledge on the cases [20]. As no clear guidelines exist, a minimum requirement for the apps was to not underestimate the urgency of the patient’s situation, regardless of whether a diagnosis was found or not. The foremost diagnoses and treatment recommendations given by the apps were assessed by the author and a score was awarded: for a correct diagnosis/treatment, 1 point; for a partially correct diagnosis or treatment, half a point, which was awarded if a correct diagnosis or treatment was not provided, but the answer would not mislead the user or underestimate the urgency (eg, if no diagnosis was found, and the app recommended visiting a real physician); for all diagnoses/treatments that did not meet these requirements, 0 points. More specifically, for glaucoma, anything less than emergency treatment would result in 0 points; for the retinal tear diagnosis, 1 point was awarded to treatment recommendation ranges of “instantly” to very few days; and for dry eyes, 0 points were awarded if treatment was deemed urgent, and half a point was awarded if trying self-treatment was not recommended before seeing a physician. A virtual anonymous patient was created to be diagnosed to prevent the potential influence of phone-based data (phone type, GPS coordinates, country, etc). Roughly two years was chosen as a deliberate time interval between tests, based on the assumption of a slow but continuous rise of the apps’ user count, and thus a slow but continuous buildup of internal data to process for possible improvements of presumed learning algorithms used by the apps. Other than the author, there were no human subjects involved in the process of this research. *P* values were calculated with a Student *t* test for independent variables using SPSS (version 16.0; IBM Corp).

### Ada

Ada is a Berlin-based app that was first tested and gained popularity on the New Zealand market in 2016 and was released more broadly afterward [21,22]. It uses a chat bot to collect data from the user, selecting symptoms from a list generated in response to the user’s free text input and subsequently asking questions that adapt to previously entered information. The resulting report may then be sent to a physician on behalf of the user.

### Babylon

Babylon is a London-based app that primarily focuses on the UK market. It started out in 2013 as a service provider for online consultations with real-life physicians, and since 2016 it has added a chat bot that presents the user with simple or multiple-choice questions for symptom assessment [23]. An explicit description of the ML algorithms that are used is not available, but judging by the publicly available information from gray literature, the use of recurrent neural networks (RNNs) for deep learning may be involved, and Python may be used as the primary programming language [24]. Ni et al mentioned the use of Bayesian networks, although a source was not given [25].

### Buoy Health

Buoy has been developed at Harvard Medical School since 2014 as a smart symptom checker with an undisclosed algorithm, supposedly relying on natural language processing (NLP)–extracted data from 18,000 clinical papers [26]. As stated by its chief executive officer and founder, Buoy Health specifically does not use decision trees but “dynamically picks” 1 of 30,000 questions based on the principle of greatest reduction of diagnostic uncertainty, which does not necessarily imply the use of neural networks. By his accounts, its diagnostic certainty is within a range of 90.9%-98%, without a detailed explanation [27].

### Your.MD

Your.MD was founded in 2012 in Oslo, Norway, and is now based in London [28,29]. Users ask free questions via its chat bot and it in turn presents simple or multiple-choice questions. The algorithms that are used are undisclosed, but judging by the publicly available information, Python may be used as a primary programming language and, according to its CEO, Bayesian networks may also be used [30].

## Results

### Ada

Ada diagnosed the angle closure glaucoma correctly in 2018 but misdiagnosed it as cluster headache in 2020 without mentioning glaucoma (this result and all following are shown in Table 1 and Table 2; for the walk-throughs with raw output data for all apps, see Multimedia Appendix 1; for an overview of the relevant results and awarded scores, see Multimedia Appendix 2).

**Table 1:** Testing of all four apps in three virtual patients with different diagnoses in 2018. Points awarded for diagnosis/treatment: (-)=0, (∗)=0.5, (•)=1.

Diagnosis	Glaucoma	Retinal tear	Dry eyes
Ada	(•)/(•)	(•)/(•)	(•)/(-)
Babylon	(-)/(•)	(-)/(-)	(-)/(-)
Buoy	(-)/(•)	(-)/(-)	(-)/(∗)
Your.MD	(•)/(•)	(•)/(•)	(•)/(•)

**Table 2:** Testing of all four apps in three virtual patients with different diagnoses in 2020. Points awarded for diagnosis/treatment: (-)=0, (∗)=0.5, (•)=1.

Diagnosis	Glaucoma	Retinal tear	Dry eyes
Ada	(-)/(-)	(•)/(•)	(•)/(∗)
Babylon	(-)/(•)	(-)/(-)	(-)/(-)
Buoy	(-)/(-)	(-)/(•)	(-)/(∗)
Your.MD	(•)/(•)	(-)/(•)	(∗)/(-)

### Babylon

In the glaucoma attack case, in both years Babylon recommended seeking emergency treatment after five questions when the user classified the pain as “severe,” with no further statement about a diagnosis. The retinal tear was not diagnosed due to “insufficient information,” and Babylon recommended referral to an online physician or real-life general practitioner. In the dry eyes case, Babylon did not state a diagnosis either and classified this as a relative emergency (same-day medical treatment recommended). There was no change in 2020.

### Buoy Health

Buoy yielded a correct diagnosis neither in 2018—although its second suggestion of “Blepharitis” could be interpreted as partly correct in the dry eyes case [31]—nor in 2020; the result for the retinal tear inquiry was far off in 2018 with “Cataract” or “Bone disease” given as possible causes. The efficiency and accuracy did not improve in 2020.

### Your.MD

Your.MD was able to output the correct diagnosis in all three tests in 2018, requiring distinctly fewer questions. In contrast to Ada, the treatment priorities were categorized correctly in all three, recognizing dry eyes as self-treatable. It was the only app to correctly identify the angle closure glaucoma, which it did in 2020; in 2018, it had only stated “Glaucoma”. In case of the retinal tear, however, it was unable to correctly identify it in 2020, while it had done so in 2018. In the dry eyes case, it changed the advice from self-treatment in 2018, which is correct, to an emergency in 2020, which is not.

### Technologies

All tested apps require an online connection to use the diagnosis function. All rely on a chat bot that is likely based on NLP and subsequently on discrete answers to questions to process user input, but they significantly differ on how the information is treated, which questions are asked, and which conclusions are drawn from the information (see Multimedia Appendix 1). There is no substantial information available on the algorithms used by the apps.

### Summary of Comparison Between Performances in 2018 and 2020

The average number of questions changed from 27.3 for Ada, 11 for Babylon, 31.3 for Buoy, and 10 for Your.MD in 2018 to 31 for Ada (*P*=.38), 9 for Babylon (*P*=.64), 30.3 for Buoy (*P*=.63), and 10.3 for Your.MD (*P*=.84) in 2020 (see Multimedia Appendix 3). In the average number of questions asked in both time periods, no difference could be found between Ada and Buoy (*P*=.41) and between Babylon and Your.MD (*P*=.93), but significant differences were found between Ada and Babylon (*P<*.001), Ada and Your.MD (*P*<.001), Babylon and Buoy (*P*<.001), and Buoy and Your.MD (*P*<.001). The scores from 2018 to 2020 changed in Ada from 3/2 to 2/1.5 (*P*=.37/.73) and in Your.MD from 3/3 to 1.5/2 (*P*=.16/.37), while Babylon and Buoy remained unchanged at 0/1 and 0/1.5, respectively (Table 1 and Table 2).

The average scores were 2.5/1.75 for Ada, 0/1 for Babylon, 0/1.5 for Buoy, and 2.25/2.5 for Your.MD, and the sums over both years were 5/3.5 for Ada, 0/2 for Babylon, 0/3 for Buoy, and 4.5/5 for Your.MD. In the sum of all points, Ada and Your.MD (*P*=.70) and Babylon and Buoy (*P*=.56) did not differ significantly, while Ada and Babylon (*P*=.02), Ada and Buoy (*P*=.03), Babylon and Your.MD (*P*=.01) and Buoy and Your.MD (*P*=.01) did.

## Discussion

During testing of the apps, some notable observations could be made about their behaviors. Ada seemed to ask redundant questions in the end, (eg, asking about eye pain when this was the primary symptom entered at the beginning). It could be speculated that this functionality serves to add input for the diagnosis to the database. Ada circumvents the common “black box” problem in ML [32] by offering a pictorial description of how many patients in 10 with the given symptoms have the suggested diagnosis. This additional information seems to indicate that by Ada’s accounts, the provided symptoms for dry eyes seemed to correspond less with the diagnosis in 2020 (“8 in 10” vs “5 in 10 people”). Here, one could speculate that there are problems incorporating the data the app accumulates over the years. Generally, the provided statistics indicate that either Bayesian probabilities are used in some way, as artificial neural network (ANN) output activities are not linked to statistical values, or the offered values are interpolated from ANN outputs. The former assumption seems to be backed up by information published by Ada Health itself, where the use of Bayesian networks is mentioned [33]. Buoy asked several questions that seemed off topic (eg, for the user’s health insurance), and in 2018 it presented users with pictures of medical conditions for comparison that may not be suitable for laymen, like testing a patellar reflex in the dry eye case or comparing one’s cornea with a microscopy picture to identify Horner-Trantas dots. In 2020, no pictures were offered for the same set of symptoms. In both years, both Babylon and Buoy failed to produce a useful diagnosis and also gave out very few treatment recommendations, with some results being very far off, such as “Bone issue” or “Non-bacterial brain inflammation” diagnoses by Buoy for the retinal tear patient, contributing to the overall result that Ada and Your.MD fared significantly better in the test than the other two. The variety of treatment recommendations given by the apps for the same starting sets of symptoms is also remarkable. Ada made it simple by generally recommending emergency care for virtually every diagnosis, which may help the manufacturers shift responsibility to the patient but counteracts the possible value of good medical advice. Babylon seemed to send any patient who chooses “severe pain” as a symptom to the emergency department, which is a good outcome for the glaucoma patient, but no diagnosis was given, and its other recommendations were very general. In 2020, Buoy gave the glaucoma patient the advice to seek medical advice within three days as its first option, followed up by “emergency treatment” as second and third options, which would confuse a real patient. Your.MD provided the most valid recommendations in this study, but also worsened on the dry eyes diagnosis from 2018 to 2020, now unnecessarily transitioning from self-treatment to emergency care.

While the number of questions the apps asked did not significantly change between the years, the temporal variances in diagnoses and treatment recommendations indicate the use of learning algorithms in all four, suggesting that the algorithms used for history-taking and diagnosis calculation are changing over time and would thus fall under the FDA’s proposed regulations for learning or “nonlocked” algorithms. In terms of their effectiveness in diagnosing ophthalmic diseases, the results were mixed with a tendency to worsening. It is noteworthy that no trend to improvement of history-taking and results could be observed at all. On the contrary, Ada and Your.MD worsened in their diagnostic outputs, while Babylon and Buoy were stable at a low level. This deterioration of diagnostic performance seems to contradict the very purpose of using “learning” algorithms in the first place and certainly justifies further inquiry. It is also notable that while two of the apps ask more questions than the other two, there seems to be no correlation between the number of questions asked and the quality of the results. On the contrary, the app with the highest overall score had the second-lowest total of questions asked. This indicates high variation in their diagnostic approaches and efficiencies, all worthy of subsequent systematic evaluation. Their algorithms are undisclosed; judging by the apps’ workflows, they all basically resemble the adaptive feedforward neural network–based mobile diagnosis engine that the author conceptualized in 2016 [34], which in its framework resembles the classic AI game “20 Questions” [35]. In both frameworks, two separate neural networks (or similar algorithms) separately calculate the current diagnosis based on the available information and the next best question based on the input up to that point. Where these examples used simple ANNs, the apps may also utilize the previously mentioned RNNs, Bayesian networks, or convolutional neural networks [15], accessed through the chat bot.

There are several limitations to this study. First, the cases that were entered included information that seemed irrelevant to the author for deciding on a suspicion of a potentially dangerous diagnosis; this includes, for example, the prevalence of diabetes, a history of smoking, and seemingly unrelated questions. It is possible, however, that to a large database that collects and sorts information without bias, answering the questions from a biased physician’s point of view might in fact mislead the algorithms. Second, the evaluation of the results is as subjective as entering the symptoms, which ultimately might test the apps’ ability to imitate a potentially flawed physician rather than whether they can correctly identify diagnoses. This could be improved by introducing systematic evaluations in the framework of a randomized controlled trial. Semigran et al had used human input and output on a randomized stack of diagnoses to assess self-diagnosis apps [7]. New methods may be necessary to investigate AI-driven apps in the future, possibly including some degree of automation considering the superhuman data storage capacities such systems can house and taking into consideration the dynamic of the algorithms. A simpler but also less systematic approach would be to include more physicians in the evaluation of the apps and average their assessments as has been done before [10]. The possibility of the manufacturers adapting to known sets of questions (eg, from this study) should also be considered upon further investigation. Third, the sample size is low. In future investigations, large-sample investigations should be preferred. Other authors like Fraser et al in 2018 have already demanded standardized and transparent procedures for examining such devices [36]. In 2019, Kelly et al advocated for a focus on peer-reviewed studies in order to increase trust in AI devices and added that the introduction of consumer-oriented technology offers the opportunity for vast prospective studies with the new collected data, provided that a sufficient level of data transparency is reached [37]. They also mentioned that an extension to the existing TRIPOD (Transparent Reporting of a multivariable prediction model for Individual Prognosis Or Diagnosis) statement, which has defined recommendations for the evaluation of diagnosis prediction since 2015 [38], is being developed to include ML algorithms [39]. In addition, the World Health Organization and the International Telecommunication Union are working on benchmarking frameworks for AI tools in health care [40]. All of these could act as guidelines for future scientific exploration of the topic, but they will require funding and manpower. The four enterprises mentioned in this paper are employing physicians, mostly with an additional formal or informal education in medical informatics or similar, at high ranks within their hierarchy, in two of them even as cofounders [41-44]. Now that this Pandora’s box of AI in the hands of patients and corporations has been opened, the question is whether this novel type of physician that supervises and administrates an automated diagnostic system will be mirrored by scientific counterparts who publicly evaluate the apps’ performances, or whether these essential data will remain undisclosed—a common practice in the commercial sector due to conflicts of interest. Considering the apps’ possible leverage and impact on public health [4], this should be in the public interest. The prospect of AI support for physicians provided by simple and accessible apps in the hands of layman users could be a golden one, as long as they actually learn and improve. Most importantly, they need to satisfy the crucial premises within the field of health care: to be efficient and safe.

## Appendix

Multimedia Appendix 1 Walkthrough through all apps and diagnoses.

Multimedia Appendix 2 Additional results tables with scores.

Multimedia Appendix 3 Additional tables (no. of questions asked, time taken).

## Abbreviations

AI: artificial intelligence
ANN: artificial neural network
FDA: US Food and Drug Administration
ML: machine learning
NLP: natural language processing
RNN: recurrent neural network
TRIPOD: Transparent Reporting of a multivariable prediction model for Individual Prognosis Or Diagnosis
