# Supplementary material for: Evaluation of Four Artificial Intelligence–Assisted Self-Diagnosis Apps on Three Diagnoses: Two-Year Follow-Up Study
Source: J Med Internet Res. 2020 Dec 4;22(12):e18097. doi: 10.2196/18097 (PMC7748958; doi:10.2196/18097)
Supplement: Multimedia Appendix 1 [file jmir_v22i12e18097_app1.docx]

# Appendix 1 – Walkthrough through all apps and diagnoses.

## Attributes used for the diagnoses and evaluations

## All diagnoses

Male patient, 40 years old, non-smoker, no pre-existing conditions.

### Glaucoma attack (primary angle-closure glaucoma, ICD-10 H40.219)

Pain in one eye of moderate to high severity, started 2 hours ago, conjunctival redness, dilated pupil, headache, blurred vision, halos around light sources.

Correct diagnose should include mention of Glaucoma or intraocular pressure, correct advice would be emergency care (within hours).

### Retinal tear (ICD-10 H33.3)

Photopsia (seeing flashes of light) in one eye for around 12 hours, no pain, no other symptoms, symptoms increase on eye movement and/or in the dark, no headache, no dizziness.

Correct diagnose should include mention of retinal involvement, including holes, tears, breaks or detachment. Correct advice would be urgent care (maximum of 24 hours).

### Dry eyes (ICD-10 H40.12)

Eye pain in both eyes for about 2 weeks, watery eyes, intermittent blurred vision, especially on reading or working on the computer, intermittent conjunctival redness, intermittent burning sensation, itchy eyes.

Correct diagnoses include dry eyes, dry eye syndrome, tear film instability, keratoconjunctivitis sicca. Possible overlap with blepharitis.

## Walkthrough for all apps on the diagnoses

Only application messages relevant for answering the following question on the pathway are noted. The apps’ statements may be shortened to leave out empty phrases. Due to their length, multiple-choice questions have been reduced to only show the selected answers (if any). All questions about symptoms not mentioned in the previous three diagnoses definitions were answered as close as possible to match a patient with the underlying diagnosis. If they were not relevant to the diagnosis from a medical point of view, or the relevance was unknown, they were answered neutrally. In Ada, the German version was used and the text subsequently translated into English.

## Ada

### Glaucoma attack; January 30^th^, 2020, 17:37-17:43

1. Who is the assessment for? – Someone else. Random name chosen, male, 40 years old, non-smoker, no history of high blood pressure, no diabetes.
2. What symptom is troubling him the most? – Painful red eye. (Being converted to “Eye pain” via search function)
3. How long has this been troubling him? – Less than one day.
4. How would he describe the intensity of the pain? – Severe.
5. Are one or both of his eyes painful? – One eye.
6. Does he have any other symptoms? – Yes. Headache.
7. How long has this been troubling him? – Less than one day.
8. Is his headache mainly located on one or both sides of the head? – One side.
9. Is it a throbbing headache? – Yes.
10. How does bending forward affect his headache? – I don’t know.
11. How would he describe the intensity of the headache? – Severe.
12. Does he have any other symptoms? Yes. Blurred vision.
13. How long has this been troubling him? – Less than one day.
14. Does the change in this vision affect one eye or both eyes? – One eye.
15. Did the change in his vision happen suddenly? – No.
16. Does he have any other symptoms? – No.
17. Are his pupils different in size? – Yes.
18. Does he have pain in the bones or skin around his eye socket? – No.
19. Is the white of his eye redder than usual? – Yes.
20. Does the redness affect one or both of his eyes? – One eye.
21. Does he have watering eyes? – Yes.
22. Does he avoid looking at bright lights due to eye painfulness? – Yes.
23. Does he have pain behind the eye? – Yes.
24. Does he feel nervous, tense or stressed out? – I don’t know.
25. Is his nose runny? – Yes.
26. How would you describe the looks of his nose discharge? – Clear.
27. Does he have a foreign body feeling in the eye? – I don’t know.
28. Does he have a swollen eyelid? – No.
29. Did he recognize an unusual discharge from his eye? – No.
30. Does he feel nauseous? – I don’t know.
31. Does he sweat a lot in his face? – No.
32. Is his eyelid hanging down? – No.
33. Is his nose painful? – No.
34. How are his symptoms changing over time? – They’re getting worse.

Diagnosis:

1. Cluster-Headache. Seek medical advice. 6 out of 10 people with the same symptoms have this condition.
2. Corneal erosion. Seek emergency care. Fewer than one out of 10 people with the same symptoms have this condition.

Total time taken: 6 minutes.

### Glaucoma attack, February 2^nd^, 2018, 16:44-16:50

1. Who is the assessment for? – Someone else. Random name chosen, male, 40 years old, non-smoker, no history of high blood pressure, no diabetes.
2. What symptom is troubling him the most? – Painful red eye. (Being converted to “Eye pain” via search function)
3. How long has this been troubling him? – Less than one day.
4. How would he describe the intensity of the pain? – Severe.
5. Are one or both of his eyes painful? – One eye.
6. Does he have any other symptoms? – Yes. Headache.
7. How long has this been troubling him? – Less than one day.
8. Is his headache mainly located on one or both sides of the head? – One side.
9. Is it a throbbing headache? – Yes.
10. How does bending forward affect his headache? – I don’t know.
11. How would he describe the intensity of the headache? – Severe.
12. Does he have any other symptoms? Yes. Blurred vision.
13. How long has this been troubling him? – Less than one day.
14. Does the change in this vision affect one eye or both eyes? – One eye.
15. Did the change in his vision happen suddenly? – No.
16. Does he have any other symptoms? – No.
17. Has he recently used contact lenses? – No.
18. Has he had an injury or surgery to the eye or the area around the eye? – No.
19. Have his pupils recently become different in size from one another? – Yes.
20. Does he have pain in the bones or skin around his eye socket? – No.
21. Does he tend to see white arcs or spots when looking at lights? – Yes.
22. Is the white of his eye redder than usual? – Yes.
23. Does the redness affect one or both of his eyes? – One eye.
24. Does he have pain behind the eye? – Yes.
25. Does he feel nauseous or as if he might vomit? – I don’t know.
26. Does he have watering eyes? – Yes.
27. Has he lost his appetite? – No.
28. Does he have swelling on any part of his face? – No.
29. Has he been vomiting? – No.
30. How are his symptoms changing over time? – They’re getting worse.

Diagnosis:

1. Acute angle closure glaucoma. Seek emergency care. 7 out of 10 people with these symptoms had this condition.
2. Cluster headache. Seek medical advice. Fewer than 1 out of 10 people with these symptoms had this condition.

Total time taken: 6 minutes.

### Retinal tear; January 30^th^, 2020, 17:57-18:02

1. Who is the assessment for? – Someone else. Random name chosen, male, 40 years old, non-smoker, no history of high blood pressure, no diabetes.
2. What symptom is troubling him the most? – Flashes in one eye. (Being converted to “Seeing flashes of light” by search function)
3. How long has this been troubling him? – Less than one day
4. Does he have any other symptoms? – No.
5. Has he had a convulsion? – No.
6. Does he have to avoid looking at bright light because it causes eye pain? – No.
7. Does he have reduced, distorted or blurred vision? – No.
8. Has he been vomiting? – No.
9. Does he involuntarily repeat movements or sounds with a constant pattern? – No.
10. Has he had an injury or surgery to the eye or the area around the eye? ­– No.
11. Does he have a headache? – No.
12. Has he recently lost consciousness? – No.
13. Does he feel excessively sleepy during daytime hours even when he has had a good night’s sleep? – No.
14. Does he get uncontrollable muscle jerks? – No.
15. Does he have any eye pain? – No.
16. Is he nauseous? – No.
17. Has he ever been diagnosed with an immune deficiency or does he take drugs influencing the immune system? – No.
18. Does he have short episodes of being unable to move his body soon after waking up or while falling asleep? – No.
19. Is he dizzy? – No.
20. Does he experience double vision? – No.
21. Does he have difficulties seeing or distinguishing between colors? – No.
22. Do straight lines or edges look bent, crooked or wavy to him? – No.
23. Okay, last question. How are his symptoms changing over time? – They’re staying about the same.

Diagnosis:

1. Retinal detachment. Seek emergency care. 5 out of 10 people with the same symptoms have this condition.
2. Migraine. Seek medical advice. 1 out of 10 people with the same symptoms have this condition.
3. Narcolepsy. Seek medical advice. Fewer than one out of 10 people with the same symptoms have this condition.
4. Carotid artery stenosis. Seek medical advice. Fewer than one out of 10 people with the same symptoms have this condition.
5. Focal seizure. Seek medical advice. Fewer than one out of 10 people with the same symptoms have this condition.

Total time taken: 5 minutes.

### Retinal tear, February 2^nd^, 2018, 17:05-17:10

1. Who is the assessment for? – Someone else. Random name chosen, male, 40 years old, non-smoker, no history of high blood pressure, no diabetes.
2. What symptom is troubling him the most? – Flashes in one eye. (Being converted to “Seeing flashes of light” by search function)
3. How long has this been troubling him? – Less than one day
4. Does he have any other symptoms? – No.
5. Has he had a convulsion? – No.
6. Has he had an injury or surgery to the eye or the area around the eye? ­– No.
7. Does he tend to suddenly freeze in the middle of completing a task or activity? – No.
8. Does he have to avoid looking at bright light because it causes eye pain? – No.
9. Has he lost his memory completely about a certain period of time or lost the ability to form new memories? – No.
10. Does he have reduced, distorted or blurred vision? – No.
11. Has he recently completely lost consciousness for a few seconds? – No.
12. Does he have a headache? – No.
13. Does he feel excessively sleepy during daytime hours even when he has had a good night’s sleep? – No.
14. Does he have any eye pain? – No.
15. Does he get uncontrollable muscle jerks? – No.
16. Has he been vomiting? – No.
17. Has he had a fall? – No.
18. Does he seem to have difficulty seeing or distinguishing between colors? – No.
19. Is he confused about where he is, what time of day it is, or who he is? – No.
20. Does he have short episodes of being unable to move his body soon after waking up or while falling asleep? – No.
21. Do straight lines or edges look bent, crooked or wavy to him? – No.
22. Does he feel extremely exhausted? – No.
23. Does he have severe difficulty adjusting his vision to low light situations? – No.
24. Does she easily get physically exhausted while exercising or during activities such as climbing stairs? – No.
25. Does he have pain behind the eye? – No.
26. Okay, last question. How are his symptoms changing over time? – They’re staying about the same.

Diagnosis:

1. Retinal detachment. Seek emergency care. 5 out of 10 people with these symptoms had this condition.
2. Non-specific photopsia. Can usually be managed at home. Fewer than one out of 10 people with these symptoms had this condition.
3. Narcolepsy. Seek medical advice. Fewer than one out of 10 people with these symptoms had this condition.

Total time taken: 5 minutes.

### Dry eyes; January 30^th^, 2020, 19:23-19:31

Who is the assessment for? – Someone else. Random name chosen, male, 40 years old, non-smoker, no history of high blood pressure, no diabetes.

1. What symptom is troubling him the most? – Eye pain. (Being converted to “Eye strain/eye pain after reading” by search function)
2. How long has this been troubling him? – One week to one month.
3. How would he describe the intensity of the pain? – Moderate.
4. Are one or both of his eyes painful? – Both eyes.
5. Does he have any other symptoms? – Yes.
6. Please name one more symptom that’s troubling him. – Tearing. (Being converted to “Watery eyes/eye tearing” by search function)
7. How long has this been troubling him? – One week to one month.
8. Does he have any other symptoms? – Yes.
9. Please name one more symptom that’s troubling him. – Blurry vision. (Being converted to “Reduced vision/blurry vision” by search function)
10. How long has this been troubling him? – One week to one month.
11. Does the change in his vision affect one eye or both eyes? – Both eyes.
12. Did the change in his vision happen suddenly? – No.
13. Does he have any other symptoms? – No.
14. Does he have a foreign body sensation in his eye? – Yes.
15. Are his eyes dry? – I don’t know.
16. Is the white of his eye redder than usual? – Yes.
17. Does the redness affect one or both of his eyes? – Both eyes.
18. Does he have to avoid looking at bright light because it causes eye pain? – Yes.
19. Did he notice an unusual discharge from his eye? – No.
20. Do his eyes feel strained and tired after looking / concentrating for a longer period on an object or an activity? – Yes.
21. Is his eyelid closing or twitching suddenly and uncontrollably? – No.
22. Is his eyelid jerking uncontrollably? – No.
23. Does one or do both eyes itch? – Yes.
24. Does he have a headache? – No.
25. Does he have any eye pain? – Yes.
26. Do his eyelids stick together? – Yes.
27. Is his eyelid particularly red? – Yes.
28. Is his red skin painful? – No.
29. Does his red skin itch? – Yes.
30. Is his eyelid sore? – No.
31. Does the skin itch in a part of his face? – No.
32. Does he have a lump in a part of his face? – No.
33. Is a part of his eye unusually white or gray? – No.
34. Does he see white arcs or spots upon looking into a light? – No.
35. Do intense light conditions lead him to having trouble seeing objects or … [rest of question could not be displayed]? – No.
36. Okay, last question. How are his symptoms changing over time? – They’re staying the same.

Diagnosis:

1. Dry eye syndrome/Keratoconjunctivitis sicca. Seek medical advice. 5 out of 10 people with the same symptoms have this condition.
2. Keratitis. Seek emergency care. One out of 10 people with the same symptoms have this condition.
3. Eye strain. Seek medical advice. Fewer than one out of 10 people with the same symptoms have this condition.
4. Blepharitis. Seek medical advice. Fewer than one out of 10 people with the same symptoms have this condition.

Total time taken: 8 minutes.

### Dry eyes, February 2^nd^, 2018, 17:33-17:38

1. Who is the assessment for? – Someone else. Random name chosen, male, 40 years old, non-smoker, no history of high blood pressure, no diabetes.
2. What symptom is troubling him the most? – Eye pain. (Being converted to “Eye strain/eye pain after reading” by search function)
3. How long has this been troubling him? – One week to one month.
4. Does he have any other symptoms? – Yes.
5. Please name one more symptom that’s troubling him. – Tearing. (Being converted to “Watery eyes/eye tearing” by search function)
6. How long has this been troubling him? – One week to one month.
7. Does he have any other symptoms? – Yes.
8. Please name one more symptom that’s troubling him. – Blurry vision. (Being converted to “Reduced vision/blurry vision” by search function)
9. How long has this been troubling him? – One week to one month.
10. Does the change in his vision affect one eye or both eyes? – Both eyes.
11. Did the change in his vision happen suddenly? – No.
12. Does he have any other symptoms? – No.
13. Are his eyes dry? – I don’t know.
14. Does he feel that there is something in his eye? – Yes.
15. Is his eyelid closing or twitching suddenly and uncontrollably? – No.
16. Does he have any eye pain? – Yes.
17. How would he describe the intensity of the eye pain? – Moderate.
18. Are one or both of his eyes painful? – Both eyes.
19. Does he have itchy eyes? – Yes.
20. Is the white of his eye redder than usual? – Yes.
21. Does the redness affect one or both of his eyes? – Both eyes.
22. Does he have to avoid looking at bright light because it causes eye pain? – Yes.
23. Do his eyelids stick together? – Yes.
24. Is his eye producing an unusual discharge? – No.
25. Is he unable to fully close his eyelids? – No.
26. Okay, last question. How are his symptoms changing over time? – They’re staying about the same.

Diagnosis:

1. Dry eye syndrome/Keratoconjunctivitis sicca. Seek emergency care. 8 out of 10 people with these symptoms had this condition.

Total time taken: 5 minutes.

## Babylon

Male patient, 40 years old, no preexisting conditions.

### Glaucoma attack, January 31^st^, 2020, 12:50-12:53

1. How can I help you? – painful red eye. => “eye pain” via symptom selector.
2. Which of these can I help you with? – Eyes are sore or itchy.
3. Which of these is the issue with his eyes? – Pain in one eye.
4. Did the eye pain start suddenly or gradually? – Gradually.
5. How bad is the pain? – Severe.

“Any pain in your eyes that is severe enough to stop you going about his day should be investigated. He should go to the nearest eye casualty as soon as you can. If there isn’t an eye casualty near him, it is important that he sees a doctor right away, so he should go to his nearest Emergency Department instead, but shouldn’t drive himself there.” Total time taken: 3 minutes.

### Glaucoma attack, March 28^th^, 2018, 17:50-17:52

1. How can I help you? – I have a painful red eye.
2. Are you experiencing any of the following? – Eye pain or eyes itchy.
3. Does any of these apply? – Pain in one eye.
4. Next question, was this a sudden or gradual change? – Symptoms started gradually.
5. Please can you describe the severity of your pain? – Severe.

“Any pain in your eyes that is severe enough to stop you going about your day should be investigated. You should go to the nearest eye casualty as soon as you can. If there isn’t an eye casualty near you, it is important that you see a doctor right away, so go to your nearest Emergency Department instead, but don’t drive yourself there.” Total time taken: 2 minutes.

### Retinal tear, January 31^st^, 2020, 12:56-13:00

1. How can I help you? – flashes of light => “Seeing flashes of light” via symptom selector.
2. Which of these can I help you with? – It’s something else
3. Has he had either yellow weeping and crustiness around the eye, or has it been watering? – Neither.
4. Has ne noticed any skin changes around his eyes? – No.
5. Has he had any of these eyelid symptoms? – None of these.
6. Does he have a lazy eye? – No.
7. Has he had any of these visual symptoms? – Seeing flashes of lights.
8. Was this a sudden or gradual change? – Sudden.
9. Does he have a headache? – No.x
10. From the options below, select all that apply to him – None of these.
11. Are his symptoms getting better, staying the same, or getting worse? – Staying the same.
12. For how long has he had symptoms? – Less than 24 hours.

“I’m sorry, I’d love to help, but on this occasion, I’d need some more information before I’m able to advise him. If he’s concerned about these symptoms, I’d recommend he speaks to a doctor.” Total time taken: 4 Minutes.

### Retinal tear, March 28^th^, 2018, 18:11-18:14

1. How can I help you? – I see flashes of light.
2. Do any of the following apply to you? – None.
3. Do any of the following apply to you? – None.
4. Next, have you noticed any skin changes around your eyes? – No.
5. Which of these are you experiencing? – None.
6. Next question, do you have a lazy eye? – No.
7. Which of these are you experiencing? – Seeing flashing lights.
8. Next, was this a sudden or gradual change? – Symptoms started suddenly.
9. Do you have a headache? – No.
10. And which of these are you experiencing? – None.
11. Okay, please can you describe the progression of your symptoms? – Stable.
12. OK. Nearly done. For how long have you had symptoms? – Less than 24 hours.

“I’m sorry, I’d love to help, but on this occasion, I’d need some more information before I’m able to advise you. Try our free Message a Clinician feature to send your symptom to a clinician, or you can speak to a GP about your problem.” Total time taken: 3 Minutes.

### Dry eyes, January 31^st^, 2020, 13:19-13:23

1. How can I help you? – Eye pain => “Eye pain” via symptom selector.
2. Which of these can I help with? - Eyes are sore or itchy.
3. Which of these is the issue with his eyes? – Eyes are itchy.
4. Has he been sneezing? – No.
5. Has he got a blocked nose? – No.
6. Has he noticed dry eyes? – Yes.
7. Has he had either yellow weeping and crustiness around the eye, or has it been watering? – Watery eye.
8. Has he developed a red eye? – Yes.
9. Has he also noticed any changes in his vision, such as flashing lights, floaters or blurred vision? – Yes.
10. For how long has he had symptoms? – Between 24 hours and 1 week.

It sounds like his symptoms require further medical assessment. I would advise him seeing someone in the next few hours. Even mild symptoms may require treatment, and he may need an eye examination so it’s important to be assessed today. He should not put this off. He can contact a GP in the first instance, but if he’s unable to do this, or his symptoms worsen in the meantime, or he is in a lot of pain or notices a drastic change in his vision, he should attend the nearest Emergency Department. He shouldn’t drive himself there.”

Total time taken: 4 minutes.

### Dry eyes, March 28^th^, 2018, 18:47-18:51

1. How can I help you? – I have eye pain.
2. Which of these are you experiencing? – Eye pain or eyes itchy.
3. Are you experiencing any of the following? – Pain in both eyes.
4. And was this a sudden or gradual change? – Symptoms started gradually.
5. Could you describe the severity of your pain? – Moderate.
6. Next question, Is there something that would make your pain worse? – None.
7. Do you have a headache? – No.
8. And have you developed a red eye? – Yes.
9. Do you wear contact lenses? – No.
10. And are your pupils equal? – Yes.
11. Is your sight deteriorating? – Yes.
12. We’re nearly done. Which of these are you experiencing? – Blurred vision.
13. Do any of the following apply to you? – None.
14. Nearly done. Have you noticed a rash on your face? – No.
15. Please can you describe the progression of your symptoms? – Stable.
16. OK, we’re nearly done. For how long have you had symptoms? – More than 1 week.

“pain, It (sic!) sounds like your symptoms require further medical assessment. I would advise seeing someone in the next few hours. Even mild symptoms may require treatment, and you may need an eye examination so it’s important to be assessed today. Please do not put this off. You can contact a GP in the first instance, but if you’re unable to do this, or your symptoms worsen in the meantime, or you are in a lot of pain or notice a drastic change in your vision, please attend the nearest Emergency Department. Don’t drive yourself there.”

Total time taken: 4 minutes.

## Buoy Health

### Glaucoma attack, January 31^st^, 2020, 14:54-15:01

1. Are you answering for yourself or someone else? – Someone else.
2. Are they male or female? – Male.
3. How old is he? – 40.
4. What symptom is bothering him the most? – Painful red eye. (Being converted to “eye redness in one eye” via search function)
5. Is his eye redness getting better or worse? – Getting worse.
6. Is his eye redness constant or come-and-go? – Constant.
7. How long has your eye been red? – 2 Hours. I’m done adding symptoms.
8. Are they thinking about seeing a medical professional for this? – Yes.
9. What care are they considering? – Urgent care.
10. Does it feel like there is something in his eye? – No.
11. Does light bother his eyes more than usual? – Yes.
12. Are his eyes watering? – Yes.
13. Does he have a runny nose? – Yes.
14. Does he feel restless or keyed up or on edge? – Unsure.
15. Any eye crusting when he wakes up? – No.
16. Do loud noises bother him more than usual? – No.
17. Do his eyelids feel sticky? – No.
18. Any issues with his eyes? – Pain.
19. Is his eye pain constant or come-and-go? – Constant.
20. Are his eyes watering? – Yes.
21. Is he tearing up in both eyes or just one? – Just one eye.
22. Does he have a headache? – Yes.
23. How severe is his headache? – Severe.
24. Has he been welding or tanning without eye protection? – No.
25. Any fever within the last week? – No.
26. Do his symptoms seem to have started or worsened after exposure to dust mites? – No.
27. Do any of these make his symptoms worse? – None of the above.
28. Is the headache…? – A new type of headache.
29. Is the eye pain affecting: - One eye.
30. What is your health insurance – Skip.
31. Do you receive health insurance through an employer (either yours or that of a family member)? – Skip.

Diagnosis:

1. Corneal abrasion. Care: Phone call or in-person visit sometime in the next 3 days.
2. Eye infection. Care: Hospital emergency room.
3. Cluster headache (first attack). Care: Hospital emergency room.

Total time taken: 7 minutes.

### Glaucoma attack, February 5^th^, 2018, 20:20-20:28

1. Are you answering for yourself or someone else? – Someone else.
2. Are they male or female? – Male.
3. How old is he? – 40.
4. What symptom is bothering him the most? – Painful red eye. (Being converted to “one red eye” via search function)
5. Is his eye redness getting better or worse? – Getting worse.
6. Is his eye redness constant or come-and-go? – Constant.
7. How long has your eye been red? – 2 Hours. I’m done adding symptoms.
8. Are they thinking about seeing a medical professional for this? – Yes.
9. What care are they considering? – Urgent care.
10. Does light bother his eyes more than usual? – Yes.
11. Are his eyes watering? – Yes.
12. Does he have a stuffy nose? – Yes.
13. Does he currently smoke? – No, never.
14. Does he have a runny nose? – No.
15. Does he suffer from sleep apnea? – No.
16. Does he feel restless or keyed up or on edge? – Unsure.
17. Is he experiencing a headache? – Yes.
18. How would he describe his headache? – This is a new type of headache.
19. Would he say his pain is – Dull or aching.
20. Does he have a sore throat? – No.
21. Does he have any of the following issues with his eye(s)? (We already know about his eye redness) – Pain.
22. Does he feel like there is something in his eye? – No.
23. Is one of his eyelids drooping like in this picture? This is called ptosis. – No.
24. Has he experienced any nausea? – Unsure.
25. Is he feeling irritable (easily made upset)? – Yes.
26. How severe is his irritability? – Moderate.
27. How severe is his headache? – Severe.
28. Has he ever been diagnosed with a specific type of headache? – None of the above.
29. Is his eye pain constant or come-and-go? – Constant.
30. Is his immune system deficient or suppressed by underlying disease or medication? – No.
31. How severe is his eye pain? – Severe.

Diagnosis: “Severe eye pain”. Recommended care: “Hospital emergency room”. Total time taken: 7 minutes.

### Retinal tear, January 31^st^, 2020, 15:07-15:14

1. Are you answering for yourself or someone else? – Someone else.
2. Are they male or female? – Male.
3. How old is he? – 40.
4. What symptom is bothering him the most? – Seeing flashes of light. => changed to “Flashing lights in vision” via symptom search.
5. How long has his vision changed? – 1 hour. – I’m done adding symptoms.
6. Are they thinking about seeing a medical professional for this? – Yes.
7. What care are they considering? – Unsure.
8. Does light bother his eyes more than usual? – No.
9. Any numbness anywhere on his body? – No.
10. Do loud noises bother him more than usual? – No.
11. Has he vomited? – No.
12. Has he experienced any nausea? – No.
13. What, if any, of these statements describe his vision changes? – None of the above.
14. Do his symptoms seem severe enough to require immediate medical help? – Yes.
15. Does he have a blood pressure machine? – No.
16. Any issues with his eyes? – None of the above.
17. Is he experiencing chills? – No.
18. Does he have a cough? – No.
19. Has he felt excessively thirsty? – No.
20. Is he so fidgety or restless that he has been moving around a lot more than usual? – No.
21. Any dizziness? – No.
22. Is he feeling less alert than normal? – No.
23. Does he have a headache? – No.
24. Any muscle weakness? – No.
25. Does he hear a ringing or whistling sound no one else hears? – No.
26. Is there a noticeable fruity odor in his breath? – No.
27. Any issues with his chest? – None of the above.
28. What is your current health insurance? – Skip.
29. Do you receive health insurance through an employer (either yours or that of a family member)? – Skip.

Diagnosis:

1. Hypertensive crisis. Care: Emergency medical service.
2. Non-bacterial brain inflammation. Care: Emergency medical service.
3. Acute close-angle glaucoma. Care: Hospital emergency room.

Total time taken: 7 minutes.

### Retinal tear, February 5^th^, 2018, 20:35-20:41

1. Are you answering for yourself or someone else? – Someone else.
2. Are they male or female? – Male.
3. How old is he? – 40.
4. What symptom is bothering him the most? – Seeing flashes of light. (This and similar terms including flashes could not be found in the symptom database; in the end, “vision change” was found to be the closest term resembling what the patient perceives, and was thus selected)
5. Are they thinking about seeing a medical professional for this? – Yes.
6. What care are they considering? – Unsure.
7. Which, if any, of these statements describe his vision changes? – Flashing lights.
8. Does light bother his eyes more than usual? – No.
9. Is he experiencing a headache? – No.
10. Is he feeling less alert than normal? – No.
11. Has he been feeling more tired than usual, lethargic or fatigued despite sleeping a normal amount? – No.
12. Has he ever been diagnosed with diabetes? – No.
13. Does he currently smoke? – No, never.
14. What is his body mass? – Average.
15. Has he been taking a corticosteroid for longer than 2 weeks? – No.
16. Does he have high blood pressure? – No.
17. Does he chew tobacco? – No.
18. Has he been on any prescription medications lately? – No.
19. Is he using a topical beta-blocker like Timolol, Levobunolol, Carteolol, Metipranolol? – No.
20. Has he experienced any nausea? – No.
21. Has he had any changes in his weight? – No.
22. Did he just suffer from a high impact injury (e.g., a fall or car accident)? – No.
23. Does he have any of the following issues with his eye(s)? – None of the above.
24. Do food or drinks get stuck when he swallows? – No.
25. About his jaw area, does he notice any of the following symptoms? – None of the above.
26. Did he get hit in the head? – No.
27. Did he faint? – No.
28. About his chest, does he notice any of the following symptoms? – None of the above.
29. Has he been experiencing dizziness? – No.

Diagnosis:

1. Cataract. Recommended care: Primary care doctor, see within 2 weeks.
2. Bone issue. Recommended care: Primary care doctor, see within 2 weeks.

Total time taken: 6 minutes.

### Dry eyes, January 31^st^, 2020, 15:20-

1. Are you answering for yourself or someone else? – Someone else.
2. Are they male or female? – Male.
3. How old is he? – 40.
4. What symptom is bothering him the most? – Eye pain. => Changed to “Pain in both eyes” via symptom search.
5. Is his eye pain getting better or worse? – Staying the same.
6. Is his eye pain constant or come-and-go? Come-and-go.
7. How long has this eye pain been going on? 2 weeks.
8. How severe is his eye pain? Moderate.
9. [Field for entering another symptom] – Blurred vision. => Changed to “Blurry vision” via symptom search.
10. How long ago did the blurry vision start? – 2 weeks. – I’m done adding symptoms.
11. Are they thinking about seeing a medical professional for this? – Yes.
12. What care are they considering? – Unsure.
13. Does he live or work in a hot and dry place? – No.
14. Has he felt excessively thirsty? – No.
15. Has he felt more tired than usual? – No.
16. Does he have an increased appetite compared to normal? – No.
17. Does it feel like there is something in his eye? – Yes.
18. Any issues with his eyes? – Redness around the eye.
19. What’s his race? – Prefer not to answer.
20. What, if any, of these statements describe his vision changes? – None of the above.
21. Any issues with peeing? – None of the above.
22. Has he been more forgetful than usual? – No.
23. Does he have a cough? – No.
24. Does he smoke or vape? – No, never.
25. What is his body mass? – Average.
26. Has he ever been diagnosed with diabetes? – No.
27. Is he sleepy during the day? – No.
28. Is the pain worse when he moves his eyes? – No.
29. Does he have a headache? – No.
30. What is your current health insurance? – Skip.
31. Do you receive health insurance through an employer (either yours or that of a family member)? – Skip.

Diagnosis:

1. Cataract. Recommended care: Primary care doctor. See within 2 weeks.
2. Non-specific eye pain. Care: Self-treatment.

Total time taken: 7 minutes.

### Dry eyes, February 5^th^, 2018, 20:51-20:59

1. Are you answering for yourself or someone else? – Someone else.
2. Are they male or female? – Male.
3. How old is he? – 40.
4. What symptom is bothering him the most? – Eye pain.
5. Eye pain can mean different things. Which are you experiencing? Mark all that apply. – He feels like there is something in the eye. His eye hurts.
6. Is his eye pain getting better or worse? – Staying the same.
7. Is his eye pain constant or come-and-go? Come-and-go.
8. How long has this eye pain been going on? 2 weeks.
9. How severe is his eye pain? Moderate.
10. [Field for entering another symptom] – Blurred vision.
11. How long ago did he notice a blurred vision? – 2 weeks.
12. – I’m done adding symptoms.
13. Are they thinking about seeing a medical professional for this? – Yes.
14. What care are they considering? – Unsure.
15. Does he live or work in a hot and dry place? – No.
16. Do his eyelids look like this? [Picture of papillary conjunctivitis shown] – No.
17. Does he have a history of any of the following? – None of the above.
18. Does anyone in his family have asthma, dermatitis, or allergies to pollen/animals/mold? – No.
19. Do his eyes look like this (known as Horner-Trantas dots)? [Picture of Horner-Trantas dots shown] – No.
20. Does he currently smoke? – No, never.
21. When he was still smoking, about how many did he smoke? – None of the above.
22. Does he have a history of heart disease? – No.
23. How much sun exposure does he normally get? – Occasional.
24. Which, if any, of these statements describe his vision changes? (We already know about his blurry vision) – None of the above.
25. While he sits on something that allows his lower legs to hang freely, have someone tap each one of his knees, just below the kneecap, as shown in the image. How do his legs react? [Picture of Patellar reflex testing shown] – Both legs kick out similarly.
26. Has anyone in his family noticed that his personality has changed recently? – No.
27. About his forearm, does he notice: - None of the above.
28. Has he noticed any of the following regarding his hands or fingers? – None of the above.
29. About his upper leg, does he notice any of the following symptoms? – None of the above.
30. Has he ever been diagnosed with diabetes? – No.
31. Does he have any of the following issues with his eye(s)? (We already know about his eye pain) – Burning sensation.
32. Has he ever suffered from a concussion or traumatic brain injury? – No.
33. Does his eye pain hurt worse when he moves his eyes? – No.
34. Is he experiencing a headache? – No.

Diagnosis:

1. Cataract. Recommended care: Primary care doctor. See within 2 weeks.
2. Inflamed eyelid (blepharitis). Recommended care: Self-treatment.
3. Type 2 diabetes. Recommended care: Primary care doctor. See within 2 weeks.

Total time taken: 8 minutes.

## Your.MD

### Glaucoma attack, January 31^st^, 2020, 19:05-19:10

1. Who is the symptom check for? – Someone else.
2. Is B male or female? – Male.
3. What year was B born in? – 1978.
4. OK, please tell me what symptoms does B have today. – Painful red eye. => understood as “eye pain and red eye/s”.
5. How long has B had these symptoms for? – Few hours.
6. Does B also have any of these symptoms today? – Red eye, severe eye pain.
7. Does B also have any of these symptoms today? – Reduced vision.
8. Has B been diagnosed with diabetes? – No.
9. Does B smoke or have they ever smoked? – No.
10. Does B also have any of these symptoms today? – None of these.
11. Does B also have any of these symptoms today? – Headache, Blurred vision.
12. Does B also have any of these symptoms today? – Headache getting worse over time.

Summary: Emergency. Even if the possible causes in this report do not seem worrying, Severe eye pain, Reduced vision, Blurred vision and Red eye/s could indicate a more serious health condition. You should call an ambulance or go to the hospital immediately. Possible causes: Acute angle closure glaucoma. Total time taken: 5 minutes.

### Glaucoma attack, February 20^th^, 2018, 18:46-18:51

1. Who is the symptom check for? – Someone else.
2. Is B male or female? – Male.
3. What year was B born in? – 1978.
4. OK, please tell me what symptoms does B have today. – Painful red eye.
5. How long has B had these symptoms for? – Few hours.
6. Does B also have any of these symptoms today? – Headache worse on looking at light, reduced vision.
7. Does B also have any of these symptoms today? – Blurred vision, headache.
8. Does B also have any of these symptoms today? – Pain on left or right side of head.
9. Does B also have any of these symptoms today? – Seeing rings around lights.

Summary: Emergency. Eye pain, Red eye/s and Headache worse on looking at light are worrying symptoms. You should call an ambulance or go to the hospital immediately. See possible causes for your reported symptoms below. Possible causes: Glaucoma. Total time taken: 5 minutes.

### Retinal tear, January 31^st^, 2020, 19:07-19:29

1. Who is the symptom check for? – Someone else.
2. Is B male or female? – Male.
3. What year was C born in? – 1978.
4. OK, please tell me what symptoms does B have today. – Seeing flashes of light.
5. How long has C had it for? – Few hours.
6. Does C also have any of these symptoms today? – None of these.
7. Has C been diagnosed with diabetes? – No.
8. Does C also have any of these symptoms today? – None of these.

Summary: I wasn’t able to find any causes for your symptoms. Please see a doctor.

Total time taken: 3 minutes

### Retinal tear, February 20^th^, 2018, 19:26-19:29

1. Who is the symptom check for? – Someone else.
2. Is B male or female? – Male.
3. What year was B born in? – 1978.
4. OK, please tell me what symptoms does B have today. – Seeing flashes of light.
5. How long has B had it for? – Few hours.
6. Does B also have any of these symptoms today? – None of these.
7. Does B also have any of these symptoms today? – None of these.
8. Does B also have any of these symptoms today? – None of these.
9. Does B also have any of these symptoms today? – None of these.

Summary: Retinal detachment. Needs emergency treatment.

Total time taken: 3 minutes.

### Dry eyes, January 31^st^, 2020, 19:27-19:32

1. Who is the symptom check for? – Someone else.
2. Is E male or female? – Male.
3. What year was E born in? – 1978.
4. OK, please tell me what symptoms does E have today. – Eye pain.
5. How long has E had it for? – Few weeks.
6. Does E also have any of these symptoms today? – None of these.
7. Does E also have any of these symptoms today? – Red eye/s.
8. Does E also have any of these symptoms today? – None of these.
9. Does E smoke or have they ever smoked? – No.
10. Does E also have any of these symptoms today? – Itchy eye/s, watery eye/s, irritated eye.
11. Does E also have any of these symptoms today? – Itchy eyes (both eyes), Sandy or gritty eyes.

Summary: Emergency. Even if the possible causes in this report do not seem worrying, Eye pain and Red eye/s could indicate a more serious health condition. You should call an ambulance or go to the hospital immediately. Possible causes: Inflammation of eyelids.

Total time taken: 5 minutes.

### Dry eyes, February 20^th^, 2018, 19:39-19:43

1. Who is the symptom check for? – Someone else.
2. Is B male or female? – Male.
3. What year was B born in? – 1978.
4. OK, please tell me what symptoms does B have today. – Eye pain.
5. How long has B had it for? – Few weeks.
6. Does B also have any of these symptoms today? – Reduced vision, Red eye/s.
7. Does B also have any of these symptoms today? – None of these.
8. Does B also have any of these symptoms today? – Blurred vision, Itchy eye/s.
9. Does B also have any of these symptoms today? –Sandy or gritty eyes.
10. Does B also have any of these symptoms today? – None of these.
11. Does B also have any of these symptoms today? – None of these.
12. Does B also have any of these symptoms today? – None of these.

Summary:

1. Inflammation of eyelids.
2. Dry eyes.

“The symptoms you reported should get better without treatment. If they continue then please come back for another consultation or see a doctor.”

Total time taken: 4 minutes.
