# Supplementary material for: Evaluation of Four Artificial Intelligence–Assisted Self-Diagnosis Apps on Three Diagnoses: Two-Year Follow-Up Study
Source: J Med Internet Res. 2020 Dec 4;22(12):e18097. doi: 10.2196/18097 (PMC7748958; doi:10.2196/18097)
Supplement: Multimedia Appendix 2 [file jmir_v22i12e18097_app2.docx]

# Appendix 2 – Additional results tables with scores.

## Ada

| Diagnosis | Glaucoma | Retinal Tear | Dry eyes | Score |
| --- | --- | --- | --- | --- |
| 2018 | Acute angle closure glaucoma **(+)**, Cluster headache (/) | Retinal detachment **(+)**, Non-specific photopsia (-), Narcolepsy (-) | Dry eye syndrome/Keratoconjunctivitis sicca **(+)** | 3 |
| 2020 | Cluster Headache **(-)**, Corneal erosion (-) | Retinal detachment **(+)**, Migraine (-), Narcolepsy (-), Carotid artery stenosis (-), Focal seizure (-) | Dry eye syndrome/Keratoconjunctivitis sicca **(+)**, Keratitis (/), Eye strain (-), Blepharitis (+) | 2 |
| Score | 1 | 2 | 2 |  |

| Treatment for: | Glaucoma | Retinal Tear | Dry eyes | Score |
| --- | --- | --- | --- | --- |
| 2018 | Seek emergency care **(+)**, Seek medical advice (-) | Seek emergency care **(+)**, Can usually be managed at home (-), Seek medical advice (/) | Seek emergency care **(-)** | 2 |
| 2020 | Seek medical advice **(-)**, Seek emergency care (+) | Seek emergency care **(+)**, Seek medical advice [4 times] (/) | Seek medical advice **(/)**, Seek emergency care (-), Seek medical advice [2 times] (/) | 1.5 |
| Score | 1 | 2 | 0.5 |  |

## Babylon

| Diagnosis | Glaucoma | Retinal Tear | Dry eyes | Score |
| --- | --- | --- | --- | --- |
| 2018 | n/a (aborted on “severe pain” as symptom) **(-)** | n/a **(-)** | n/a **(-)** | 0 |
| 2020 | n/a (aborted on “severe pain” as symptom) **(-)** | n/a **(-)** | n/a **(-)** | 0 |
| Score | 0 | 0 | 0 |  |

| Treatment for: | Glaucoma | Retinal Tear | Dry eyes | Score |
| --- | --- | --- | --- | --- |
| 2018 | Emergency care [paraphrased] **(+)** | „try Babylon Messaging To Doctors system, or eventually speak to a GP“ [paraphrased] **(-)** | “see doctor within few hours” **(-)** | 1 |
| 2020 | Emergency care [paraphrased] **(+)** | „eventually speak to a doctor“ [paraphrased] **(-)** | “see doctor within few hours” **(-)** | 1 |
| Score | 2 | 0 | 0 |  |

## Buoy

| Diagnosis | Glaucoma | Retinal Tear | Dry eyes | Score |
| --- | --- | --- | --- | --- |
| 2018 | Severe eye pain **(-)** | Cataract **(-)**, Bone issue (-) | Cataract **(-)**, Inflamed eyelid (blepharitis) (+), Type 2 diabetes (-) | 0 |
| 2020 | Corneal abrasion **(-)**, Eye infection (-), Cluster headache (first attack) (-) | Hypertensive Crisis **(-)**, Non-bacterial brain inflammation (-), Acute angle closure glaucoma (/) | Cataract **(-)**, Non-specific eye pain (-) | 0 |
| Score | 0 | 0 | 0 |  |

| Treatment for: | Glaucoma | Retinal Tear | Dry eyes | Score |
| --- | --- | --- | --- | --- |
| 2018 | Hospital emergency room **(+)** | Primary care doctor, see within 2 weeks **(-)** [2 times] | Primary care doctor, see within 2 weeks **(/)** [repeated later], Self-treatment (+) | 1.5 |
| 2020 | Phone call or in-person visit sometime in the next 3 days **(-)**, Hospital emergency room [2 times] | Emergency medical service [2 times] **(+)**, Hospital emergency room (+) | Primary care doctor, see within 2 weeks **(/)**, Self-treatment (+) | 1.5 |
| Score | 1 | 1 | 1 |  |

## Your.MD

| Diagnosis | Glaucoma | Retinal Tear | Dry eyes | Score |
| --- | --- | --- | --- | --- |
| 2018 | Glaucoma **(+)** | Retinal detachment **(+)** | Inflammation of eyelids, Blepharitis **(+)** | 3 |
| 2020 | Acute angle closure glaucoma **(+)** | n/a **(-)** | Inflammation of eyelids **(/)** | 1.5 |
| Score | 1 | 1 | 1.5 |  |

| Treatment for: | Glaucoma | Retinal Tear | Dry eyes | Score |
| --- | --- | --- | --- | --- |
| 2018 | Call an ambulance or go to hospital immediately **(+)** | Needs emergency treatment **(+)** | The symptoms […] should get better without treatment […] **(+)** | 3 |
| 2020 | Call an ambulance or go to hospital immediately **(+)** | Please see a doctor **(+)** | Emergency. You should call an ambulance or go to the hospital immediately. **(-)** | 2 |
| Score | 2 | 2 | 1 |  |
