# Supplementary material for: Evaluation of Four Artificial Intelligence–Assisted Self-Diagnosis Apps on Three Diagnoses: Two-Year Follow-Up Study
Source: J Med Internet Res. 2020 Dec 4;22(12):e18097. doi: 10.2196/18097 (PMC7748958; doi:10.2196/18097)
Supplement: Multimedia Appendix 3 [file jmir_v22i12e18097_app3.docx]

# Appendix 3: Additional tables (No. of questions asked, time taken).

## Ada

| Diagnosis | Glaucoma | Retinal Tear | Dry eyes | Average |
| --- | --- | --- | --- | --- |
| Questions asked 2018 | 30 | 26 | 26 | 27.3 |
| Time taken 2018 [min] | 6 | 5 | 5 | 5.3 |
| Questions asked 2020 | 34 | 23 | 36 | 31 |
| Time taken 2020 [min] | 6 | 5 | 8 | 6.3 |

## Babylon

| Diagnosis | Glaucoma | Retinal Tear | Dry eyes | Average |
| --- | --- | --- | --- | --- |
| Questions asked 2018 | 5 | 12 | 16 | 11 |
| Time taken 2018 [min] | 2 | 3 | 4 | 3 |
| Questions asked 2020 | 5 | 12 | 10 | 9 |
| Time taken 2020 [min] | 3 | 4 | 4 | 3.7 |

## Buoy

| Diagnosis | Glaucoma | Retinal Tear | Dry eyes | Average |
| --- | --- | --- | --- | --- |
| Questions asked 2018 | 31 | 29 | 34 | 31.3 |
| Time taken 2018 [min] | 7 | 7 | 8 | 7.3 |
| Questions asked 2020 | 31 | 29 | 31 | 30.3 |
| Time taken 2020 [min] | 7 | 7 | 7 | 7 |

## Your.MD

| Diagnosis | Glaucoma | Retinal Tear | Dry eyes | Average |
| --- | --- | --- | --- | --- |
| Questions asked 2018 | 9 | 9 | 12 | 10 |
| Time taken 2018 [min] | 5 | 3 | 4 | 4 |
| Questions asked 2020 | 12 | 8 | 11 | 10.3 |
| Time taken 2020 [min] | 5 | 3 | 5 | 4.3 |
